# The role of gadolinium-based contrast agents in magnetic resonance imaging structured reporting and data systems (RADS)

**DOI:** 10.1007/s10334-023-01113-y

**Published:** 2023-09-13

**Authors:** Marco Parillo, Carlo Augusto Mallio, Aart J. Van der Molen, Àlex Rovira, Ilona A. Dekkers, Uwe Karst, Gerard Stroomberg, Olivier Clement, Eliana Gianolio, Aart J. Nederveen, Alexander Radbruch, Carlo Cosimo Quattrocchi, ESMRMB-GREC Working Group

**Affiliations:** 1 Fondazione Policlinico Universitario Campus Bio-Medico, Via Alvaro del Portillo, 200, 00128 Rome, Italy; 2 Research Unit of Diagnostic Imaging and Interventional Radiology, Department of Medicine and Surgery, Università Campus Bio-Medico di Roma, Via Alvaro del Portillo, 21, 00128 Rome, Italy; 3 Department of Radiology, C-2S, Leiden University Medical Center, Albinusdreef 2, 2333 ZA Leiden, The Netherlands; 4 Section of Neuroradiology, Department of Radiology, Hospital Universitari Vall d’Hebron, Universitat Autònoma de Barcelona, Barcelona, Spain; 5 Institute of Inorganic and Analytical Chemistry, University of Münster, Corrensstr. 48, 48149 Münster, Germany; 6 RIWA-Rijn-Association of River Water Works, Groenendael 6, 3439 LV Nieuwegein, The Netherlands; 7 Service de Radiologie, Université de Paris, AP-HP, Hôpital Européen Georges Pompidou, DMU Imagina, 20 Rue LeBlanc, 75015 Paris, France; 8 Department of Molecular Biotechnologies and Health Science, University of Turin, Via Nizza 52, 10125 Turin, Italy; 9 Department of Radiology and Nuclear Medicine, Amsterdam UMC, University of Amsterdam, Amsterdam, Netherlands; 10 Department of Neuroradiology, University Hospital Bonn, Rheinische Friedrich-Wilhelms-Universität Bonn, 53127 Bonn, Germany; 11 Centre for Medical Sciences-CISMed, University of Trento, Via S. Maria Maddalena 1, 38122 Trento, Italy

**Keywords:** Gadolinium, RADS, Reporting and data systems, MRI, Contrast media

## Abstract

Among the 28 reporting and data systems (RADS) available in the literature, we identified 15 RADS that can be used in Magnetic Resonance Imaging (MRI). Performing examinations without using gadolinium-based contrast agents (GBCA) has benefits, but GBCA administration is often required to achieve an early and accurate diagnosis. The aim of the present review is to summarize the current role of GBCA in MRI RADS. This overview suggests that GBCA are today required in most of the current RADS and are expected to be used in most MRIs performed in patients with cancer. Dynamic contrast enhancement is required for correct scores calculation in PI-RADS and VI-RADS, although scientific evidence may lead in the future to avoid the GBCA administration in these two RADS. In Bone-RADS, contrast enhancement can be required to classify an aggressive lesion. In RADS scoring on whole body-MRI datasets (MET-RADS-P, MY-RADS and ONCO-RADS), in NS-RADS and in Node-RADS, GBCA administration is optional thanks to the intrinsic high contrast resolution of MRI. Future studies are needed to evaluate the impact of the high T1 relaxivity GBCA on the assignment of RADS scores.

**Supplementary Information:**

The online version contains supplementary material available at 10.1007/s10334-023-01113-y.

## Introduction

In recent years, there has been a large-scale dissemination of clinical reporting guidelines in radiology in the form of Reporting and Data Systems (RADS), which have been proposed as standardized systems for imaging reporting to minimize variations and ambiguous terminology, facilitating images interpretation and outcomes monitoring [1]. Many RADS have been developed by the American College of Radiology (ACR), starting in 1993 with the Breast Imaging-Reporting and Data System (BI-RADS), while some RADS were developed by other groups [2, 3]. The RADS are both modality and technique specific. The role of gadolinium-based contrast agents (GBCA) is still a topic of strong debate, as evidenced by the increasing literature and discussions at international conferences, including the ISMRM-ESMRMB hot topic debate in the 2022 joint annual meeting [4], on the role of GBCA and opportunities for reduced dose and non-contrast imaging. In fact, GBCA administration is often required to achieve an early and accurate diagnosis [5–8]; in addition, the use of contrast agent can improve the diagnostic ability of less experienced readers. On the other hand, performing examinations without using GBCA has a number of benefits such as: reduced contrast agent-related operational issues, including less pre-MRI patient documentation, blood tests, and safety checks (e.g., allergies and renal function assessments); no concerns regarding potential contrast agent side effects (e.g., contrast extravasation, hematoma, nephrogenic systemic fibrosis, gadolinium deposition, and allergic reactions) [9–11]; no contrast agent-related infrastructure (e.g., additional staff, inserting and removing cannulas, and preparing contrast injectors) with cost savings in terms of decreased material and infrastructural use; shortened examination times [12, 13]. In addition, reducing the use of GBCA will reduce their unintended impact on aquatic ecosystems and drinking water resources [14, 15].

The scope of the present review is to summarize the current role of GBCA only in clinical reporting guidelines for MRI that have adopted the “RADS” approach, focusing on three specific questions per each RADS: A. what is the scope of the scoring system; B. how are GBCA used in the scoring system; C. what is the impact of GBCA enhancement on the scoring. Table 1 summarizes the main RADS currently in use in MRI and the role of GBCA.

**Table 1:** Summary of the main RADS currently used in MRI and the role of the contrast agent for each, listed in alphabetical order

Magnetic resonance imaging reporting and data system	Clinical indication	Scope	Contrast enhancement
American College of Radiology Breast Imaging-Reporting and Data System (ACR BI-RADS)	Breast cancer	Diagnosis	Dynamic contrast enhancement is required
American College of Radiology Liver Imaging-Reporting and Data System (ACR LI-RADS)	Liver cancer	Diagnosis	Multiphase contrast enhancement is required
American College of Radiology Neck Imaging-Reporting and Data System (ACR NI-RADS)	Head and neck cancer	Surveillance	Contrast enhancement is required
American College of Radiology Ovarian-Adnexal Reporting and Data System (ACR O-RADS)	Ovarian-adnexal mass	Diagnosis	Dynamic contrast enhancement is required; if not available contrast enhancement should be used
American College of Radiology Prostate Imaging-Reporting and Data System (ACR PI-RADS)	Prostate cancer	Diagnosis	Dynamic contrast enhancement is required; greater evidence is needed to define which patient groups can safely avoid gadolinium administration
Bone Reporting and Data System (Bone‑RADS)	Bone lesion	Diagnosis	Contrast enhancement is often required
Bone Tumor Imaging-Reporting and Data System (BTI-RADS)	Bone lesion	Diagnosis	Contrast enhancement is required
Brain Tumor-Reporting and Data System (BT-RADS)	Brain cancer	Surveillance	Contrast enhancement is required
METastasis Reporting and Data System for Prostate Cancer (MET-RADS-P)	Bone and nodal disease in advanced prostate cancer	Diagnosis and surveillance	Contrast enhancement is optional
Myeloma Response Assessment and Diagnosis System (MY-RADS)	Multiple myeloma	Diagnosis and surveillance	Contrast enhancement is optional
Node Reporting and Data System 1.0 (Node-RADS)	Lymph nodes in cancer	Diagnosis	Contrast enhancement is optional
Neuropathy Score Reporting and Data System (NS-RADS)	Peripheral neuropathy	Diagnosis	Contrast enhancement is optional
Oncologically Relevant Findings Reporting and Data System (ONCO-RADS)	Cancer screening	Diagnosis	Contrast enhancement is optional
Osseous Tumor Reporting and Data System (OT-RADS)	Bone lesion	Diagnosis	Contrast enhancement is required
Vesical Imaging-Reporting and Data System (VI-RADS)	Bladder cancer	Diagnosis	Dynamic contrast enhancement is required; recent studies highlight the possibility of avoiding GBCA administration

## Literature search strategy

**Fig. 1:**
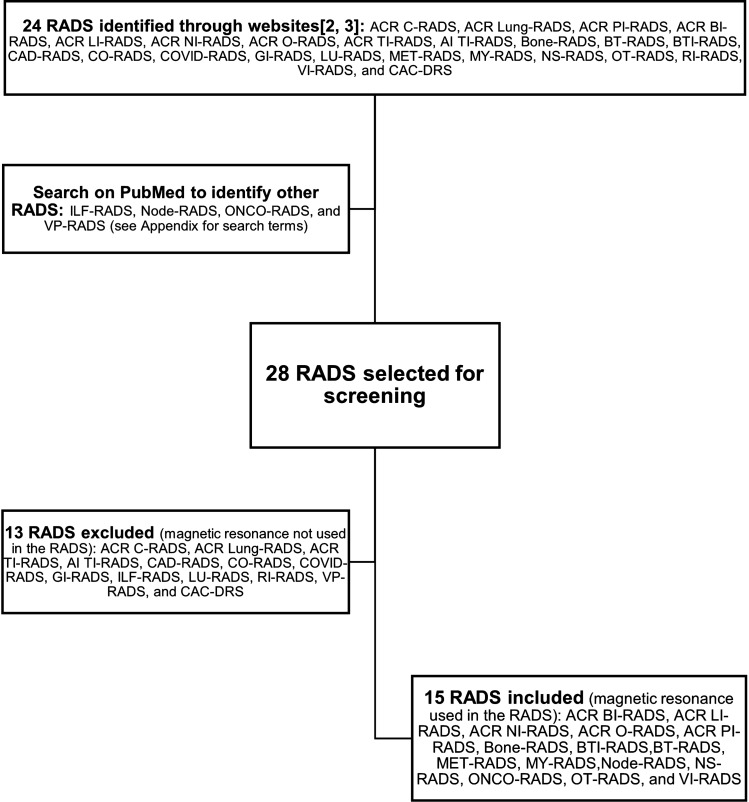
Flow diagram of the search strategy and study selection

We identified 24 RADS through websites [2, 3]; a subsequent search on PubMed (timeframe between January 1, 2005 and April 29, 2023) was performed, identifying four additional RADS in the literature. Thirteen RADS were excluded, because they did not involve MRI. In the final analysis, we found 15 RADS suitable for use in MRI out of the 28 RADS described in the literature. See Fig. 1 for the flow diagram of the search strategy and study selection and Appendix for details on the search terms used on PubMed. We read the original articles for each latest version of RADS.

Five RADS were endorsed and developed by the ACR: American College of Radiology Breast Imaging-Reporting and Data System (ACR BI-RADS) 5th edition [16, 17], American College of Radiology Liver Imaging-Reporting and Data System (ACR LI-RADS) version 2018 [18], American College of Radiology Neck Imaging-Reporting and Data System (ACR NI-RADS) [19–22], American College of Radiology Ovarian-Adnexal Reporting and Data System (ACR O-RADS) [23–25], and American College of Radiology Prostate Imaging-Reporting and Data System (ACR PI-RADS) version 2.1 [26, 27].

Ten RADS were proposed by other scientific groups: Bone Reporting and Data System (Bone‑RADS) [28], Bone Tumor Imaging-Reporting and Data System (BTI-RADS) [29], Brain Tumor Reporting and Data System (BT-RADS) [30, 31], METastasis Reporting and Data System for Prostate Cancer (MET-RADS-P) [32], Myeloma Response Assessment and Diagnosis System (MY-RADS) [33], Node Reporting and Data System 1.0 (Node-RADS) [34], Neuropathy Score Reporting and Data System (NS-RADS) [35], Oncologically Relevant Findings Reporting and Data System (ONCO-RADS) [36], Osseous Tumor-Reporting and Data System (OT-RADS) [37], and Vesical Imaging-Reporting and Data System (VI-RADS) [38].

## Current RADS used in MRI

### ACR BI-RADS 5th edition [16, 17, 39, 40]

A. It is a risk assessment and a standardized system of reporting breast pathology that relates categories to management recommendations. It applies to mammography, contrast-enhanced mammography, ultrasound, and contrast-enhanced MRI.
B. T1-weighted dynamic contrast enhancement (DCE) imaging (GBCA dosage of 0.1 mmol/kg body weight) with a maximum acquisition time of 60–120 s per sequence of both breasts is included in the standard MRI protocol combined with bright-fluid and T1-weighted pre-contrast sequences, preferably with fat suppression; subtraction imaging and kinetic curve assessment may be desired. An additional suggested sequence is diffusion weighted imaging (DWI).
C. Enhancement is essential in the assessment of background parenchymal enhancement and in the description of any area of abnormal enhancement, including focus, mass, and non-mass enhancement for the final assignment of the score (from 0 to 6).

### ACR LI-RADS v2018 [18, 41–43]

A. It is a risk assessment for hepatocellular carcinoma (HCC) and a standardized system of reporting imaging findings in liver lesions of patients with risk factors for HCC, that relates categories to management recommendations. It also allows to assess the response of HCC to locoregional treatment. It applies to contrast-enhanced computed tomography (CT), contrast-enhanced ultrasound, ultrasound, and contrast-enhanced MRI.
B. Multiphase post-contrast T1-weighted imaging (GBCA dosage of 0.1 mmol/kg body weight) [arterial phase (late arterial phase strongly preferred), portal venous phase, delayed phase, and hepatobiliary phase if using gadoxetate disodium (GBCA dosage of 0.025 mmol/kg body weight)] is combined with unenhanced T1-weighted in- and opposed-phase imaging and T2-weighted sequences. Subtraction imaging may be desired. Additional suggested sequences are DWI and 1- to 3-h hepatobiliary phase if using gadobenate dimeglumine (GBCA dosage of 0.1 mmol/kg body weight).
C. Enhancement is essential in the assessment of some major features: arterial phase hyperenhancement, non-peripheral washout, and the presence of an enhancing capsule. Contrast enhancement is also needed for the investigation of some ancillary features: corona enhancement, transitional phase hypointensity, hepatobiliary phase hypointensity or isointensity, mosaic appearance, and parallel blood pool enhancement. In addition, administration of contrast agent allows identification of the targetoid dynamic enhancement (favorable for LR-malignant but not HCC specific) and enhancement of a venous thrombus. Thus, multiphase imaging is critical for the final assignment of the category (from negative to 5 including LR-tumor in vein and LR-malignant but not HCC specific) and to highlight any residual tumor in the post-treatment LI-RADS assignment.

### ACR NI-RADS [19–22]

A. It is a structured head and neck surveillance reporting system after treatment with categories tied to follow up recommendations. Originally developed for surveillance using contrast-enhanced CT with or without PET; NI-RADS can also be applied to contrast-enhanced MRI, mostly for the evaluation of perineural spread.
B. A post-contrast T1-weighted sequence (GBCA dosage of 0.1 mmol/kg body weight) is included in the standard MRI protocol combined with T1-weighted and T2-weighted pre-contrast sequences; an additional suggested sequence is DWI.
C. Enhancement is essential in the evaluation of the primary site and the neck (nodal assessment), in the assignment of all categories (from 1 to 4).

### ACR O-RADS [23–25, 44, 45]

A. It is a risk assessment and a standardized system of reporting ovarian-adnexal pathology that relates categories to management recommendations. It applies to ultrasound and contrast-enhanced MRI.
B. A DCE-MRI should be performed using a T1-weighted sequence before and after intravenous administration of GBCA (GBCA dosage of 0.1 mmol/kg body weight) to evaluate the time-intensity curves (temporal resolution < 15 s). If DCE-MRI is not possible, then non-DCE-MRI can be performed as a pre- and post-contrast T1-weighted sequence performed 30–40 s after the end of contrast agent injection. Imaging protocol should include at least T2-weighted sequences without fat saturation, T1-weighted in- and opposed-phase images, and DWI.
C. Enhancement is essential in the identification of solid tissue within an adnexal lesion, that raises the suspicion of malignancy. Thus, post-contrast imaging is critical for the final assignment of the category (from 0 to 5) and, in particular, DCE is the key in discriminating between categories 3, 4, and 5 based on the time-intensity curves relative to the outer myometrium.

### ACR PI-RADS v2.1 [26, 27, 46, 47]

A. The scope is to improve detection, localization, characterization, and risk stratification in patients with suspected prostate cancer in treatment naïve glands.
B. A DCE with rapid T1-weighted gradient echo sequence (temporal resolution: ≤ 15 s) before, during, and after the intravenous administration of GBCA (GBCA dosage of 0.1 mmol/kg body weight) is currently included in the multi-parameter MRI protocol. Fat suppression or subtraction techniques are proposed to improve the detection of enhancement. Imaging protocol should include at least also a pre-contrast T1-weighted sequence.
C. Although DCE is a component of the multiparametric MRI prostate examination, its role in the determination of PI-RADS v2.1 score is secondary to T2-weighted images and DWI. A positive DCE (defined as focal and earlier than or contemporaneously with enhancement of adjacent normal prostatic tissues and corresponds to suspicious finding on T2-weighted images and/or DWI) upgrades a DWI + PI-RADS 3 in the peripheral zone to PI-RADS 4 (3 + 1). DCE may improve the sensitivity and detection of cancer in both the peripheral and transitional zones, especially when DWI is degraded by artifacts or when less experienced readers are reporting.

### Bone‑RADS [28]

A. It is a risk assessment and a standardized system of reporting incidental solitary bone lesions that relates categories to management recommendations. It applies to CT and MRI.
B. Post-contrast imaging is often required to exclude a malignancy, but its evaluation is secondary to pre-contrast T1- and T2-weighted sequences (the latter also with fat suppression).
C. In the presence of a T1 hyperintense solitary bone lesion without macroscopic intralesional fat, the type of contrast enhancement allows to classify the lesion as Bone-RADS 1 (none or thin peripheral enhancement) or Bone-RADS 4 (nodular and/or central enhancement). In the presence of a T1 hypointense solitary bone lesion, the evidence of solid mass enhancement categorizes the lesion as Bone-RADS 4.

### BTI-RADS [29]

A. It is a classification system for solitary bone lesions based on various benign and malignant indicators. It applies to CT and MRI.
B. A T1-weighted post-contrast sequence, 5 min after GBCA administration (GBCA dosage of 0.1 mmol/kg body weight), is included in the MRI protocol along with at least two orthogonal T2-weighted with fat suppression images.
C. The absence of contrast enhancement is included in the “benign indicators”, while the type of contrast enhancement (homogenous or heterogeneous) is listed in the “indeterminate features”.

### BT-RADS [30, 31, 48]

A. It is a structured primary brain tumor surveillance reporting system with categories tied to management recommendations using contrast-enhanced MRI.
B. A T1-weighted post-contrast sequence (GBCA dosage of 0.1 mmol/kg body weight) is included in the standard MRI protocol combined with T2-weighted Fluid Attenuated Inversion Recovery (FLAIR) sequences; additional suggested sequences are dynamic susceptibility contrast perfusion (to evaluate relative cerebral blood volume) and DWI.
C. Enhancement is essential for the overall assessment of the examination, especially in the assignment of categories 3b, 3c, and 4.

### MET-RADS-P [32]

A. The scope is to promote standardization in the reporting of whole body-MRI (WB-MRI) in advanced prostate cancer at the baseline study and in the follow-up of the patient, evaluating response to treatment in metastatic disease. The main purpose is the evaluation of bone and nodal disease, while a more extensive assessment should be used for patients with established visceral disease. The assessment of the prostate or prostatectomy bed is not an essential requirement of the scoring system.
B. Post-contrast imaging is not mandatory in the “core protocol”, when the aim is to obtain information on bone and nodal disease. The main sequences used are: T1-weighted [Turbo Spin Echo (TSE) and Dixon], Short Tau Inversion Recovery (STIR), and DWI.
C. GBCA can be used in a more comprehensive assessment, including dedicated prostate or brain studies.

### MY-RADS [33, 49]

A. The scope is to promote standardization in the reporting of WB-MRI in myeloma at the baseline study and in the follow-up of the patient, evaluating response to treatment. The main purpose is the evaluation of bone marrow, while a more extensive assessment should be used for appraisal of soft tissue, extramedullary disease, or for those patients in whom serial tumor response assessments (including clinical trials) are planned.
B. Post-contrast imaging is not mandatory in the “core clinical protocol”, when the aim is to obtain information on bone marrow involvement. The main sequences used are: T1-weighted (TSE and Dixon), STIR, and DWI.
C. GBCA can be used in a more comprehensive assessment, including soft tissue or extramedullary disease evaluation.

### Node-RADS [34]

A. The scope is to stratify the risk of having cancer involvement in regional and distant lymph nodes, increasing consensus among radiologists for primary staging and in response assessment settings. It applies to CT and MRI.
B. Post-contrast imaging is not mandatory for MRI because of the intrinsic high soft-tissue contrast, while the use of contrast agents is required for CT scans. The sequences to be evaluated are those where the assessment of the criteria “size” and “configuration” succeeds best.
C. Although it is not strictly necessary for the nodal evaluation, GBCA are often essential in tumor staging and follow-up.

### NS-RADS [35, 50]

A. The scope is to improve the reporting and evaluation of peripheral neuropathy on MRI.
B. Post-contrast imaging is not mandatory. The main sequences used are fat-suppressed T2-weighted sequences and either proton density weighted or T1-weighted sequences.
C. Enhancement can be useful especially when there is suspicion of nerve neoplasia (subclass N of the scoring system).

### ONCO-RADS [36]

A. The scope is to stratify the risk of having malignant tumors in individuals undergoing WB-MRI for cancer screening in the general population and in predisposition syndromes.
B. Post-contrast imaging is not mandatory for WB-MRI and should be avoided in general population cancer screening. The standard protocol is based on the following sequences: T1-weighted (TSE, Dixon and gradient echo for the lung), STIR, T2-weighted, DWI, and T2-FLAIR (for the brain).
C. GBCA should be used in a more comprehensive assessment when there is a requirement for investigating additional body parts (e.g., soft-tissue mass or breast evaluations) or for brain evaluation in patients with Li-Fraumeni syndrome, neurofibromatosis, constitutional mismatch repair deficiency syndrome, and hereditary retinoblastoma.

### OT-RADS [37]

A. The scope is to standardize the classification of osseous tumors to facilitate the differentiation between benign and malignant lesions, achieving good-to-excellent interreader agreement. It applies to MRI.
B. A post-contrast T1-weighted fat-suppressed sequence (GBCA dosage of 0.1 mmol/kg body weight) is required in a “complete MRI study”. Imaging protocol also includes unenhanced T1-weighted images, fat-suppressed T2-weighted or STIR sequences, and DWI.
C. Contrast enhancement features are needed for the final assignment of the score (from II to V). An incomplete examination, for example without GBCA administration, is classified as OT-RADS 0 and requires further imaging.

### VI-RADS [38, 51–53]

A. It is a systematic approach to reporting MRI of bladder cancer defining, in particular, the risk of muscle layer invasion.
B. A T1-weighted DCE imaging (GBCA dosage of 0.1 mmol/kg body weight) is required before and at 30 s after the beginning of injection and is followed by the same sequences four-to-six times every 30 s to depict the early enhancement of the inner layer followed by tumor enhancement. The late phase is useless in local staging, because signal contrast among the inner and outer layers and tumor decreases. Imaging protocol must include at least T2-weighted sequences without fat saturation and DWI. Quantitative measurements, such as apparent diffusion coefficient measurement and perfusion curves, are optional.
C. DCE is a key component for the final assignment of VI-RADS score (from 1 to 5). The final category is first based on T2-weighted sequences for the morphology. The presence of definitive muscular invasion is decided using DWI and DCE-MRI (especially when DWI is tainted by artifacts).

## Discussion

This overview suggests that GBCA are today required in most of the current RADS and, thus, are expected to be used in most MRIs performed in patients with cancer. Several authors, different from those who have proposed RADS, have analyzed the possible role of unenhanced MRI (uMRI) in RADS protocols also with the support of artificial intelligence, aiming to carry gadolinium-analog information. As an example, regarding BI-RADS, unenhanced MRI with STIR and DWI sequences had the same diagnostic performance compared to contrast-enhanced MRI in patients with BI-RADS 0 (lesions that need further investigation for complete analysis) [54]. Another study, comparing uMRI + Digital Breast Tomosynthesis (DBT) with DCE-MRI, concluded that DCE-MRI was the most sensitive imaging technique in breast cancer preoperative assessment despite the good accuracy of uMRI + DBT [55]. In a 3.0 T scanner setting, diagnostic performance and interreader agreement of both uMRI and DCE-MRI were high, with inferior lesion conspicuity and lower interreader agreement of uMRI [56].

Regarding the O-RADS, a recent study has highlighted that the morphological and qualitative DWI assessment by gynecological radiologists could be an alternative when intravenous contrast agent and a dynamic curve assessment for the formal O–RADS score cannot be provided [57].

In recent years, many studies have investigated the possible role of uMRI in PI-RADS, based on T2-weighted and DWI sequences (so-called biparametric MRI) in patients with treatment naïve prostate cancer. Most works compared uMRI versus DCE-MRI in screening and evaluating prostatic gland, showing that contrast enhancement had no or only a marginal effect on the diagnostic performance for detecting clinically significant cancers. However, the role of DCE-MRI in increasing the score from PI-RADS 3 to PI-RADS 4, the diagnostic impact of GBCA enhancement when T2-weighted and DWI sequences are degraded by artifacts, or the importance of contrast agents in helping radiologists with less experience were not often evaluated [58]. A simplified PI-RADS based on biparametric MRI has also been proposed, with the scope to assist radiologists and urologists in the detection and management of prostatic cancer [59]. Additionally, artificial intelligence tools have been exponentially developed in the setting of prostate cancer diagnosis by taking advantage of the opportunities of uMRI. A recent review summarized the role of machine-learning or deep-learning in biparametric prostate MRI, showing promising results in terms of cancer detection and differentiation from non-cancerous tissue. However, the authors pointed out that there was still great variability between reports and only a few multicenter studies were available [60–62]. Therefore, if machine-learning could help in avoiding the systematic use of GBCA in prostate MRI and the use of deep learning-based softwares could improve reporting times, the clinical applicability of these approaches still requires more robust validation across scanner vendors, field strengths, and institutions. The PI-RADS Committee concluded that the biparametric MRI requires optimal image acquisition and data interpretation, the possibility of instituting patient recalls or on-table monitoring of images when there is insufficient quality and in indeterminate cases. As an alternative approach, it remains desirable to tailor the need for GBCA-enhanced MRI according to patient risk. In fact, men at intermediate risk might undergo contrast-enhanced MRI as the default approach. The PI-RADS Committee underlined the need for further higher quality data before issuing evidence-based recommendations about unenhanced MRI as an initial diagnostic approach for prostate cancer workup [13].

Similar to PI-RADS, an alternative “biparametric” protocol has been proposed in VI-RADS, consisting only of T2-weighted and DWI sequences without the use of GBCA, employing 1.5 and 3.0 T MRI scanners, with a comparable diagnostic accuracy to the standard MRI protocol for the detection of muscle-invasive bladder cancer [63–66]; a meta-analysis confirmed these results [67]. In these cases, a denoising deep-learning reconstruction could significantly improve the diagnostic accuracy of T2-weighted sequences [68]. On the other hand, the use of DCE-MRI could provide additional value to the radiomics-based identification of muscle-invasive cancer [69].

Although these studies show promising results, the use of artificial intelligence applied to the field of RADS in clinical practice is still uncommon.

Another aspect to consider relates to the increasing use of high T1 relaxivity contrast agents, which allow reducing the GBCA dose without compromising image quality and diagnostic accuracy. Gadopiclenol 0.5 mmol/ml is a new GBCA approved for clinical use in September 2022 by the Food and Drug Administration at a dosage of 0.05 mmol/kg body weight [70]. It is based on a pyclen macrocyclic structure, offering good stabilities (thermodynamic and kinetic) and high r1 relaxivity (about twofold that of other macrocyclic GBCA). These characteristics confer a double benefit: use half of the standard dose to get the same efficacy and use the standard dose to get a higher enhancement [71]. Another high T1 relaxivity contrast agent, named gadoquatrane, is currently under development with a preclinical profile characterized by favorable physicochemical properties and the same pharmacokinetic profile as currently used GBCA; thus, gadoquatrane represents an excellent candidate for further clinical development [72]. In most oncology imaging, and therefore also in the RADS for MRI that we have listed, 0.1 mmol/kg body weight of GBCA is used, delivered at a flow rate of 2–4 mL/s [73]. In particular, the signal change observed in DCE-MRI depends on the concentration of the contrast agent applied; at low-to-modest GBCA concentrations T1 shortening leads to signal increases, while at high GBCA concentrations, signal losses due to T2* shortening occur, affecting, for example, the time–intensity curves [74]. Therefore, the use of these new high-relaxivity GBCA could affect the longitudinal evaluation of oncological patients when examinations performed with different classes of contrast agent are compared. Future studies are needed to evaluate the impact of these new GBCA on the assignment of RADS scores, especially when DCE is required.

## Conclusion

- Currently GBCA administration plays a key role in most MRI RADS.
- Dynamic contrast enhancement is required for score calculation in ACR PI-RADS and VI-RADS, although scientific evidence may lead in the future to avoid the GBCA administration in these two RADS also with the aid of artificial intelligence tools.
- In Bone-RADS contrast enhancement is often required to classify a lesion as Bone-RADS 4.
- In RADS where WB-MRI is applied (MET-RADS-P, MY-RADS, and ONCO-RADS), in NS-RADS and in Node-RADS, GBCA is optional due to the intrinsic high contrast resolution of MRI.
- Future studies are needed to evaluate the impact of the next-generation high T1 relaxivity GBCA on the assignment of RADS scores.

### Supplementary Information

Below is the link to the electronic supplementary material.

Supplementary file1 (DOCX 13 KB)

## Data Availability

No new data are associated with this review article.

## Abbreviations

ACR: American College of Radiology
BI-RADS: Breast imaging reporting and data system
Bone-RADS: Bone reporting and data system
BTI-RADS: Bone tumor imaging reporting and data system
BT-RADS: Brain tumor reporting and data system
CT: Computed tomography
DBT: Digital breast tomosynthesis
DCE: Dynamic contrast enhancement
DWI: Diffusion weighted imaging
ESMRMB: European Society for Magnetic Resonance in Medicine and Biology
FLAIR: Fluid attenuated inversion recovery
GBCA: Gadolinium-based contrast agent
GREC: Gadolinium Research and Education Committee
HCC: Hepatocellular carcinoma
ISMRM: International Society for Magnetic Resonance in Medicine
LI-RADS: Liver imaging reporting and data system
MET-RADS-P: METastasis reporting and data system for prostate cancer
MRI: Magnetic resonance imaging
MY-RADS: Myeloma response assessment and diagnosis system
NI-RADS: Neck imaging reporting and data system
Node-RADS: Node Reporting and Data System 1.0
NS-RADS: Neuropathy score reporting and data system
ONCO-RADS: Oncologically relevant findings reporting and data system
O-RADS: Ovarian-adnexal reporting and data system
OT-RADS: Osseous tumor reporting and data system
PI-RADS: Prostate imaging reporting and data system
RADS: Reporting and data systems
STIR: Short tau inversion recovery
TSE: Turbo spine echo
uMRI: Unenhanced MRI
VI-RADS: Vesical imaging reporting and data system
WB-MRI: Whole body-magnetic resonance imaging

## References

[1] An JY, Unsdorfer KML, Weinreb JC (2019). BI-RADS, C-RADS, CAD-RADS, LI-RADS, Lung-RADS, NI-RADS, O-RADS, PI-RADS, TI-RADS: reporting and data systems. Radiographics.

[2] Bell DJ (2005) Reporting and data systems (disambiguation). In: Radiology reference article. Radiopaedia.org. Radiopaedia. 10.53347/rID-76158

[3] Reporting and Data Systems. https://www.acr.org/Clinical-Resources/Reporting-and-Data-Systems. Accessed 14 Dec 2022

[4] Quattrocchi CC, Agarwal N, Taso M, Dekkers IA (2022). Report on the ISMRM-ESMRMB 2022 hot topic debate on the future of gadolinium as a contrast agent. MAGMA.

[5] Parillo M, Vaccarino F, Quattrocchi CC (2023). Imaging findings in a case of leptomeningeal myelomatosis, a rare but critical central nervous system complication of multiple myeloma. Neuroradiol J.

[6] Parillo M, Bernetti C, Altomare C, Beomonte Zobel B, Quattrocchi CC (2023). Extrahepatic abscess and dropped gallstones: a case report and a narrative review of an unusual delayed complication of laparoscopic cholecystectomy. Acta Chir Belg.

[7] De Stefano D, Parillo M, Garipoli A, Beomonte Zobel B (2021). Imaging findings in a case of myo-pericarditis associated with SARS CoV-2 disease. J Cardiol Cases.

[8] Parillo M, Vertulli D, Mallio CA, Quattrocchi CC (2023). Imaging findings in carcinomatous encephalitis secondary to malignant melanoma. Egypt J Neurol Psychiatry Neurosurg.

[9] Parillo M, Mallio CA, Van der Molen AJ, Rovira À, Ramalho J, Ramalho M, Gianolio E, Karst U, Radbruch A, Stroomberg G, Clement O, Dekkers IA, Nederveen AJ, Quattrocchi CC, ESMRMB-GREC Working Group (2023). Skin toxicity after exposure to gadolinium-based contrast agents in normal renal function, using clinical approved doses: current status of preclinical and clinical studies. Invest Radiol.

[10] Parillo M, Sapienza M, Arpaia F, Magnani F, Mallio CA, DʼAlessio P, Quattrocchi CC (2019). A structured survey on adverse events occurring within 24 hours after intravenous exposure to gadodiamide or gadoterate meglumine: a controlled prospective comparison study. Invest Radiol.

[11] Quattrocchi CC, Parillo M, Spani F, Landi D, Cola G, Dianzani C, Perrella E, Marfia GA, Mallio CA (2023). Skin thickening of the scalp and high signal intensity of dentate nucleus in multiple sclerosis: association with linear versus macrocyclic gadolinium-based contrast agents administration. Invest Radiol.

[12] ESUR Guidelines 10 (2018). https://www.esur.org/wp-content/uploads/2022/03/ESUR-Guidelines-10_0-Final-Version.pdf. Accessed 10 May 2023

[13] Schoots IG, Barentsz JO, Bittencourt LK, Haider MA, Macura KJ, Margolis DJA, Moore CM, Oto A, Panebianco V, Siddiqui MM, Tempany C, Turkbey B, Villeirs GM, Weinreb JC, Padhani AR (2021). PI-RADS committee position on MRI without contrast medium in biopsy-naive men with suspected prostate cancer: narrative review. AJR Am J Roentgenol.

[14] Birka M, Wehe CA, Hachmöller O, Sperling M, Karst U (2016). Tracing gadolinium-based contrast agents from surface water to drinking water by means of speciation analysis. J Chromatogr A.

[15] Schmidt K, Bau M, Merschel G, Tepe N (2019). Anthropogenic gadolinium in tap water and in tap water-based beverages from fast-food franchises in six major cities in Germany. Sci Total Environ.

[16] American College of Radiology Committee on BI-RADS® (2013). https://www.acr.org/-/media/ACR/Files/RADS/BI-RADS/MRI-Reporting.pdf

[17] Morris EA, Comstock CE, Lee CH (2013). ACR BI-RADS® magnetic resonance imaging ACR BI-RADS® atlas, breast imaging reporting and data system.

[18] American College of Radiology Committee on LI-RADS® (2018). https://www.acr.org/-/media/ACR/Files/RADS/LI-RADS/LI-RADS-2018-Core.pdf. Accessed 10 May 2023

[19] Aiken AH, Hudgins PA (2018). Neck imaging reporting and data system. Magn Reson Imaging Clin N Am.

[20] Aiken AH, Farley A, Baugnon KL, Corey A, El-Deiry M, Duszak R, Beitler J, Hudgins PA (2016). Implementation of a novel surveillance template for head and neck cancer: neck imaging reporting and data system (NI-RADS). J Am Coll Radiol.

[21] Aiken AH, Rath TJ, Anzai Y, Branstetter BF, Hoang JK, Wiggins RH, Juliano AF, Glastonbury C, Phillips CD, Brown R, Hudgins PA (2018). ACR neck imaging reporting and data systems (NI-RADS): a white paper of the ACR NI-RADS committee. J Am Coll Radiol.

[22] American College of Radiology Committee on NI-RADS^TM^ (2021). https://www.acr.org/-/media/ACR/Files/RADS/NI-RADS/ACR-NI-RADS-MRI-Table.pdf. Accessed 10 May 2023

[23] Sadowski EA, Thomassin-Naggara I, Rockall A, Maturen KE, Forstner R, Jha P, Nougaret S, Siegelman ES, Reinhold C (2022). O-RADS MRI risk stratification system: guide for assessing adnexal lesions from the ACR O-RADS committee. Radiology.

[24] Reinhold C, Rockall A, Sadowski EA, Siegelman ES, Maturen KE, Vargas HA, Forstner R, Glanc P, Andreotti RF, Thomassin-Naggara I (2021). Ovarian-adnexal reporting lexicon for MRI: a white paper of the ACR ovarian-adnexal reporting and data systems MRI committee. J Am Coll Radiol.

[25] American College of Radiology Committee on O-RADS^TM^ (2021). https://www.acr.org/-/media/ACR/Files/RADS/O-RADS/O-RADS-MR-Lexicon-Terms-Table.pdf. Accessed 10 May 2023

[26] Turkbey B, Rosenkrantz AB, Haider MA, Padhani AR, Villeirs G, Macura KJ, Tempany CM, Choyke PL, Cornud F, Margolis DJ, Thoeny HC, Verma S, Barentsz J, Weinreb JC (2019). Prostate imaging reporting and data system version 2.1: 2019 update of prostate imaging reporting and data system version 2. Eur Urol.

[27] American College of Radiology Committee on PI-RADS^TM^ (2019). https://www.acr.org/-/media/ACR/Files/RADS/PI-RADS/PIRADS-V2-1.pdf. Accessed 10 May 2023

[28] Chang CY, Garner HW, Ahlawat S, Amini B, Bucknor MD, Flug JA, Khodarahmi I, Mulligan ME, Peterson JJ, Riley GM, Samim M, Lozano-Calderon SA, Wu JS (2022). Society of Skeletal Radiology-white paper. Guidelines for the diagnostic management of incidental solitary bone lesions on CT and MRI in adults: bone reporting and data system (Bone-RADS). Skeletal Radiol.

[29] Ribeiro GJ, Gillet R, Hossu G, Trinh J-M, Euxibie E, Sirveaux F, Blum A, Teixeira PAG (2021). Solitary bone tumor imaging reporting and data system (BTI-RADS): initial assessment of a systematic imaging evaluation and comprehensive reporting method. Eur Radiol.

[30] Weinberg BD, Gore A, Shu H-KG, Olson JJ, Duszak R, Voloschin AD, Hoch MJ (2018). Management-based structured reporting of posttreatment glioma response with the brain tumor reporting and data system. J Am Coll Radiol.

[31] Home-Brain Tumor Reporting and Data System (BT-RADS). In: Brain tumor rads (BT-Rads). https://btrads.com/. Accessed 16 Oct 2022

[32] Padhani AR, Lecouvet FE, Tunariu N, Koh D-M, De Keyzer F, Collins DJ, Sala E, Schlemmer HP, Petralia G, Vargas HA, Fanti S, Tombal HB, de Bono J (2017). METastasis reporting and data system for prostate cancer: practical guidelines for acquisition, interpretation, and reporting of whole-body magnetic resonance imaging-based evaluations of multiorgan involvement in advanced prostate cancer. Eur Urol.

[33] Messiou C, Hillengass J, Delorme S, Lecouvet FE, Moulopoulos LA, Collins DJ, Blackledge MD, Abildgaard N, Østergaard B, Schlemmer H-P, Landgren O, Asmussen JT, Kaiser MF, Padhani A (2019). Guidelines for acquisition, interpretation, and reporting of whole-body MRI in myeloma: myeloma response assessment and diagnosis system (MY-RADS). Radiology.

[34] Elsholtz FHJ, Asbach P, Haas M, Becker M, Beets-Tan RGH, Thoeny HC, Padhani AR, Hamm B (2021). Introducing the node reporting and data system 1.0 (Node-RADS): a concept for standardized assessment of lymph nodes in cancer. Eur Radiol.

[35] Chhabra A, Deshmukh SD, Lutz AM, Fritz J, Andreisek G, Sneag DB, Subhawong T, Singer AD, Wong PK, Thakur U, Pandey T, Chalian M, Mogharrabi BN, Guirguis M, Xi Y, Ahlawat S (2022). Neuropathy score reporting and data system: a reporting guideline for MRI of peripheral neuropathy with a multicenter validation study. AJR Am J Roentgenol.

[36] Petralia G, Koh D-M, Attariwala R, Busch JJ, Eeles R, Karow D, Lo GG, Messiou C, Sala E, Vargas HA, Zugni F, Padhani AR (2021). Oncologically relevant findings reporting and data system (ONCO-RADS): guidelines for the acquisition, interpretation, and reporting of whole-body MRI for cancer screening. Radiology.

[37] Chhabra A, Gupta A, Thakur U, Pezeshk P, Dettori N, Callan A, Xi Y, Weatherall P (2021). Osseous tumor reporting and data system-multireader validation study. J Comput Assist Tomogr.

[38] Panebianco V, Narumi Y, Altun E, Bochner BH, Efstathiou JA, Hafeez S, Huddart R, Kennish S, Lerner S, Montironi R, Muglia VF, Salomon G, Thomas S, Vargas HA, Witjes JA, Takeuchi M, Barentsz J, Catto JWF (2018). Multiparametric magnetic resonance imaging for bladder cancer: development of VI-RADS (vesical imaging-reporting and data system). Eur Urol.

[39] Spick C, Bickel H, Polanec SH, Baltzer PA (2018). Breast lesions classified as probably benign (BI-RADS 3) on magnetic resonance imaging: a systematic review and meta-analysis. Eur Radiol.

[40] Li J, Zheng H, Cai W, Wang Y, Zhang H, Liao M (2020). Subclassification of BI-RADS 4 magnetic resonance lesions: a systematic review and meta-analysis. J Comput Assist Tomogr.

[41] Shin J, Lee S, Yoon JK, Son WJ, Roh YH, Chung YE, Choi J-Y, Park M-S (2022). Diagnostic performance of LI-RADS v2018 versus KLCA-NCC 2018 criteria for hepatocellular carcinoma using magnetic resonance imaging with hepatobiliary agent: a systematic review and meta-analysis of comparative studies. Gut Liver.

[42] Kim Y-Y, Lee S, Shin J, Son WJ, Roh YH, Hwang JA, Lee JE (2022). Diagnostic performance of CT versus MRI liver imaging reporting and data system category 5 for hepatocellular carcinoma: a systematic review and meta-analysis of comparative studies. Eur Radiol.

[43] van der Pol CB, McInnes MDF, Salameh J-P, Chernyak V, Tang A, Bashir MR, LI-RADS IPD Group, LI-RADS IPD Group Collaborators (2022). Impact of reference standard on CT, MRI, and contrast-enhanced US LI-RADS diagnosis of hepatocellular carcinoma: a meta-analysis. Radiology.

[44] Rizzo S, Cozzi A, Dolciami M, Del Grande F, Scarano AL, Papadia A, Gui B, Gandolfo N, Catalano C, Manganaro L (2022). O-RADS MRI: a systematic review and meta-analysis of diagnostic performance and category-wise malignancy rates. Radiology.

[45] Vara J, Manzour N, Chacón E, López-Picazo A, Linares M, Pascual MÁ, Guerriero S, Alcázar JL (2022). Ovarian adnexal reporting data system (O-RADS) for classifying adnexal masses: a systematic review and meta-analysis. Cancers (Basel).

[46] Wen J, Ji Y, Han J, Shen X, Qiu Y (2022). Inter-reader agreement of the prostate imaging reporting and data system version v2.1 for detection of prostate cancer: a systematic review and meta-analysis. Front Oncol.

[47] Oerther B, Engel H, Bamberg F, Sigle A, Gratzke C, Benndorf M (2022). Cancer detection rates of the PI-RADSv2.1 assessment categories: systematic review and meta-analysis on lesion level and patient level. Prostate Cancer Prostatic Dis.

[48] Parillo M, Mallio CA, Pileri M, Dirawe D, Romano A, Bozzao A, Weinberg B, Quattrocchi CC (2023). Interrater reliability of brain tumor reporting and data system (BT-RADS) in the follow up of adult primary brain tumors: a single institution experience in Italy. Quant Imaging Med Surg.

[49] Rata M, Blackledge M, Scurr E, Winfield J, Koh D-M, Dragan A, Candito A, King A, Rennie W, Gaba S, Suresh P, Malcolm P, Davis A, Nilak A, Shah A, Gandhi S, Albrizio M, Drury A, Roberts S, Jenner M, Brown S, Kaiser M, Messiou C (2022). Implementation of whole-body MRI (MY-RADS) within the OPTIMUM/MUKnine multi-centre clinical trial for patients with myeloma. Insights Imaging.

[50] Chhabra A, Deshmukh SD, Lutz AM, Fritz J, Sneag DB, Mogharrabi B, Guirguis M, Andreisek G, Xi Y, Ahlawat S (2022). Neuropathy score reporting and data system (NS-RADS): MRI reporting guideline of peripheral neuropathy explained and reviewed. Skeletal Radiol.

[51] Jazayeri SB, Dehghanbanadaki H, Hosseini M, Taghipour P, Bazargani S, Thomas D, Feibus A, Sarabchian E, Bacchus MW, Di Valerio EA, Bandyk M, Balaji KC (2022). Inter-reader reliability of the vesical imaging-reporting and data system (VI-RADS) for muscle-invasive bladder cancer: a systematic review and meta-analysis. Abdom Radiol (NY).

[52] Del Giudice F, Flammia RS, Pecoraro M, Moschini M, D’Andrea D, Messina E, Pisciotti LM, De Berardinis E, Sciarra A, Panebianco V (2022). The accuracy of vesical imaging-reporting and data system (VI-RADS): an updated comprehensive multi-institutional, multi-readers systematic review and meta-analysis from diagnostic evidence into future clinical recommendations. World J Urol.

[53] Woo S, Panebianco V, Narumi Y, Del Giudice F, Muglia VF, Takeuchi M, Ghafoor S, Bochner BH, Goh AC, Hricak H, Catto JWF, Vargas HA (2020). Diagnostic performance of vesical imaging reporting and data system for the prediction of muscle-invasive bladder cancer: a systematic review and meta-analysis. Eur Urol Oncol.

[54] Zhang R, Xu M, Zhou C, Ding X, Lu H, Ge M, Du L, Bu Y (2022). The value of noncontrast MRI in evaluating breast imaging reporting and data system category 0 lesions on digital mammograms. Quant Imaging Med Surg.

[55] Rizzo V, Moffa G, Kripa E, Caramanico C, Pediconi F, Galati F (2021). Preoperative staging in breast cancer: intraindividual comparison of unenhanced MRI combined with digital breast tomosynthesis and dynamic contrast enhanced-MRI. Front Oncol.

[56] Baltzer PAT, Bickel H, Spick C, Wengert G, Woitek R, Kapetas P, Clauser P, Helbich TH, Pinker K (2018). Potential of noncontrast magnetic resonance imaging with diffusion-weighted imaging in characterization of breast lesions: intraindividual comparison with dynamic contrast-enhanced magnetic resonance imaging. Invest Radiol.

[57] Sahin H, Panico C, Ursprung S, Simeon V, Chiodini P, Frary A, Carmo B, Smith J, Freeman S, Jimenez-Linan M, Bolton H, Haldar K, Ang JE, Reinhold C, Sala E, Addley H (2021). Non-contrast MRI can accurately characterize adnexal masses: a retrospective study. Eur Radiol.

[58] Belue MJ, Yilmaz EC, Daryanani A, Turkbey B (2022). Current status of biparametric MRI in prostate cancer diagnosis: literature analysis. Life (Basel).

[59] Scialpi M, Scialpi P, Martorana E, Torre R, Improta A, Aisa MC, D’Andrea A, Di Blasi A (2021). Simplified PI-RADS (S-PI-RADS) for biparametric MRI to detect and manage prostate cancer: What urologists need to know. Turk J Urol.

[60] Michaely HJ, Aringhieri G, Cioni D, Neri E (2022). Current value of biparametric prostate MRI with machine-learning or deep-learning in the detection, grading, and characterization of prostate cancer: a systematic review. Diagnostics (Basel).

[61] Cipollari S, Pecoraro M, Forookhi A, Laschena L, Bicchetti M, Messina E, Lucciola S, Catalano C, Panebianco V (2022). Biparametric prostate MRI: impact of a deep learning-based software and of quantitative ADC values on the inter-reader agreement of experienced and inexperienced readers. Radiol Med.

[62] Cuocolo R, Cipullo MB, Stanzione A, Ugga L, Romeo V, Radice L, Brunetti A, Imbriaco M (2019). Machine learning applications in prostate cancer magnetic resonance imaging. Eur Radiol Exp.

[63] Watanabe M, Taguchi S, Machida H, Tambo M, Takeshita Y, Kariyasu T, Fukushima K, Shimizu Y, Okegawa T, Fukuhara H, Yokoyama K (2022). Clinical validity of non-contrast-enhanced VI-RADS: prospective study using 3-T MRI with high-gradient magnetic field. Eur Radiol.

[64] Aslan S, Cakir IM, Oguz U, Bekci T, Demirelli E (2022). Comparison of the diagnostic accuracy and validity of biparametric MRI and multiparametric MRI-based VI-RADS scoring in bladder cancer; is contrast material really necessary in detecting muscle invasion?. Abdom Radiol (NY).

[65] Delli Pizzi A, Mastrodicasa D, Marchioni M, Primiceri G, Di Fabio F, Cianci R, Seccia B, Sessa B, Mincuzzi E, Romanelli M, Castellan P, Castellucci R, Colasante A, Schips L, Basilico R, Caulo M (2021). Bladder cancer: do we need contrast injection for MRI assessment of muscle invasion? A prospective multi-reader VI-RADS approach. Eur Radiol.

[66] Elshetry ASF, El-Fawakry RM, Hamed EM, Metwally MI, Zaid NA (2022). Diagnostic accuracy and discriminative power of biparametric versus multiparametric MRI in predicting muscle-invasive bladder cancer. Eur J Radiol.

[67] Ye L, Chen Y, Xu H, Xie H, Yao J, Liu J, Song B (2022). Biparametric magnetic resonance imaging assessment for detection of muscle-invasive bladder cancer: a systematic review and meta-analysis. Eur Radiol.

[68] Taguchi S, Tambo M, Watanabe M, Machida H, Kariyasu T, Fukushima K, Shimizu Y, Okegawa T, Yokoyama K, Fukuhara H (2021). Prospective validation of vesical imaging-reporting and data system using a next-generation magnetic resonance imaging scanner-is denoising deep learning reconstruction useful?. J Urol.

[69] Liu Y, Xu X, Wang H, Liu Y, Wang Y, Dong Q, Li Z, Guo Y, Lu H (2022). The additional value evaluation of tri-parametric MRI in identifying muscle-invasive status in bladder cancer. Acad Radiol.

[70] FDA Gadopiclenol (2022). https://www.accessdata.fda.gov/drugsatfda_docs/label/2022/216986s000lbl.pdf. Accessed 10 May 2023

[71] Lancelot E, Raynaud J-S, Desché P (2020). Current and future MR contrast agents: seeking a better chemical stability and relaxivity for optimal safety and efficacy. Invest Radiol.

[72] Lohrke J, Berger M, Frenzel T, Hilger C-S, Jost G, Panknin O, Bauser M, Ebert W, Pietsch H (2022). Preclinical profile of gadoquatrane: a novel tetrameric, macrocyclic high relaxivity gadolinium-based contrast agent. Invest Radiol.

[73] Shukla-Dave A, Obuchowski NA, Chenevert TL, Jambawalikar S, Schwartz LH, Malyarenko D, Huang W, Noworolski SM, Young RJ, Shiroishi MS, Kim H, Coolens C, Laue H, Chung C, Rosen M, Boss M, Jackson EF (2019). Quantitative imaging biomarkers alliance (QIBA) recommendations for improved precision of DWI and DCE-MRI derived biomarkers in multicenter oncology trials. J Magn Reson Imaging.

[74] Petralia G, Summers PE, Agostini A, Ambrosini R, Cianci R, Cristel G, Calistri L, Colagrande S (2020). Dynamic contrast-enhanced MRI in oncology: how we do it. Radiol Med.

