# Supplementary material for: The role of gadolinium-based contrast agents in magnetic resonance imaging structured reporting and data systems (RADS)
Source: MAGMA. 2023 Sep 13;37(1):15–25. doi: 10.1007/s10334-023-01113-y (PMC10876744; doi:10.1007/s10334-023-01113-y)
Supplement: Supplementary file 1 — Supplementary file1 (DOCX 13 KB) [file 10334_2023_1113_MOESM1_ESM.docx]

**PubMed Advanced Search Builder**

- (Reporting and Data Systems) OR (RADS) OR (-RADS) OR (Reporting & Data Systems)
- (-RADS[Title]) NOT (C-RADS) NOT (Lung-RADS) NOT (PI-RADS) NOT (BI-RADS) NOT (LI-RADS) NOT (NI-RADS) NOT (O-RADS) NOT (TI-RADS) NOT (Bone-RADS) NOT (BT-RADS) NOT (BTI-RADS) NOT (CAD-RADS) NOT (CO-RADS) NOT (COVID-RADS) NOT (GI-RADS) NOT (LU-RADS) NOT (MET-RADS) NOT (MY-RADS) NOT (NS-RADS) NOT (OT-RADS) NOT (RI-RADS) NOT (VI-RADS) NOT (CAC-DRS)
